# The Impact of Copay Accumulators and Maximizers on Treatment Patterns, Adherence, and Costs Among Patients with Major Depressive and Bipolar Disorders Treated with Branded Therapies

**DOI:** 10.3390/jmahp13040055

**Published:** 2025-11-07

**Authors:** Onur Baser, Katarzyna Rodchenko, Heidi C. Waters, Matthew Sullivan, Lixuan Wu, Shuangrui Chen, Madeline Shurtleff, Cynthia Bigley, Rashmi Patel

**Affiliations:** 1 Graduate School of Public Health, City University of New York (CUNY), New York, NY 10027, USA; 2 Department of Economics, Boğaziçi University, Istanbul 34342, Türkiye; 3 Columbia Data Analytics, 145 Hudson Street, Suite 205, New York, NY 10013, USA; rkatarzyna@cdanyc.com (K.R.);; 4 Otsuka Pharmaceutical Development & Commercialization, Inc., 508 Carnegie Center Drive, Princeton, NJ 08054, USAmatthew.sullivan@otsuka-us.com (M.S.);; 5 Department of Psychiatry, The Old Schools, University of Cambridge, Trinity Ln, Cambridge CB2 1TN, UK

**Keywords:** copay accumulator, copay maximizer, major depressive disorder, bipolar disorder, atypical antipsychotics, antidepressants, adherence, out-of-pocket costs

## Abstract

Copay accumulator (CA) and copay maximizer (CM) programs in the United States, which prevent manufacturer copay assistance from counting toward deductibles or out-of-pocket (OOP) maximums, are increasingly used, raising concerns about costs and outcomes for patients with major depressive disorder (MDD) or bipolar disorder (BPD) treated with branded atypical antipsychotics (AAPs) and/or antidepressants (ADs). This retrospective claims study used Kythera commercial data (2020–2024) in the United States to identify adults with MDD or BPD who had at least 1 diagnosis and one branded AAP or AD prescription between 2021 and 2023, requiring 12 months’ continuous enrollment pre- (2020–2021) and post-index (2023–2024) and at least three months of post-index branded medication use. This retrospective claims study used Kythera commercial data (2020–2024) to identify adults with MDD or BPD who had at least one diagnosis and one branded AAP or AD prescription between 2021 and 2023, requiring 12 months’ continuous enrollment pre- and post-index and at least 3 months of post-index branded medication use. Patients were stratified into CA, CM, or standard copay plan (SCP) cohorts, and propensity score matching was used to compare treatment patterns and costs. Both CA and CM groups had significantly higher median OOP costs than SCPs (e.g., $75/$60 vs. $16 for MDD+AAP; *p* < 0.0001), and higher pharmacy costs among adherent patients. CA patients had poorer adherence and persistence, shorter treatment duration, and higher discontinuation and abandonment rates than SCPs. These findings highlight higher OOP burden and adherence challenges with CA and CM programs, underscoring the need for careful benefit design for US mental health patients.

## 1. Introduction

Benefit designs are the set of rules that structure health insurance plans, determining how consumers access healthcare services, which services are covered, which providers can be used, and the cost-sharing amounts (such as deductibles, copayments ‘copays’, and coinsurance) for which the consumer is responsible [1]. These designs have evolved from simple, broad coverage to complex, cost-sharing models aimed at controlling costs and incentivizing value [2].

The evolution of group health insurance in the United States began in the 1920s and 1930s with Blue Cross and Blue Shield introducing prepaid hospital and physician services, characterized by minimal cost-sharing and broad coverage that included most hospital and physician services [3]. In the postwar era from the 1940s to the 1960s, employer-sponsored insurance expanded rapidly as wage controls during World War II prompted employers to offer health benefits to attract workers [4]. The introduction of Medicare and Medicaid in 1965 mirrored private insurance structures but focused on the elderly and low-income populations [5]. From the 1970s to the 1990s, the emergence of health maintenance organizations and preferred provider organizations brought more managed care, with networks, utilization review, and increased cost-sharing through deductibles, copayments, and coinsurance, while benefit designs began to include exclusions for certain services and experimental treatments [6]. Since the 2000s, benefit designs have grown more complex, incorporating tiered networks, prior authorizations, and specialty drug management, while high-deductible health plans paired with Health Savings Accounts became popular, shifting more cost responsibility to consumers and introducing value-based insurance design to incentivize high-value care and discourage low-value services [7]. The evolution of benefit design was not occurring in isolation; rather, it closely paralleled significant changes in the treatment landscape. Over time, there has been a marked increase in the availability of therapies for long-term and chronic disorders, as opposed to the historical focus on short-duration treatments for acute conditions. Many of these newer therapies, particularly biologics and monoclonal antibodies, are substantially more expensive than previous generations of treatments, whether for acute or chronic indications. This shift toward high-cost, long-term therapies has placed considerable financial pressure on healthcare plans, prompting the adoption of more restrictive and complex benefit designs, shifted more costs to patients.

However, research has consistently demonstrated that higher out-of-pocket (OOP) costs are associated with decreased medication adherence, poorer disease control, and increased rates of hospitalization [8]. In response to these challenges, pharmaceutical manufacturers frequently offer copay assistance programs intended to improve patient access to medications by offsetting the cost-sharing obligations imposed by insurance plans. Manufacturer-sponsored copay assistance provided $23 billion in patient cost support in 2023, reflecting a $5 billion increase over the previous year [9]. Despite the intended patient benefit, some payers contend that these programs allow patients to circumvent formulary designs, potentially leading to the selection of higher-cost brand-name medications over less expensive generic alternatives. As a result, starting from mid-2010s, insurers and pharmacy benefit managers implemented copay adjustment programs (CAPs), including copay accumulator (CA) and copay maximizer (CM) programs, which redirect manufacturer assistance funds from patients to insurers or third-party administrators [10]. In 2023 alone, such programs accounted for $4.8 billion in redirected copay assistance [9].

Copay accumulator programs operate by excluding manufacturer assistance from counting toward a patient’s deductible or OOP maximum; once the assistance is exhausted, patients remain responsible for their full deductible and cost-sharing requirements, thereby transferring the financial benefit to the insurance plan [11]. In contrast, CM programs require patients to enroll with a third-party administrator, which determines the maximum available manufacturer assistance and advises the insurance plan to adjust monthly copays accordingly, ensuring the plan captures the full value of the assistance [11]. While CMs may minimize OOP costs for patients on specified drugs, the assistance provided does not contribute toward deductibles or OOP maximums. These approaches have been described as “cost-shifting programs,” as they enable payers to capture a substantial portion of the financial support originally intended for patients [11]. Therefore, these payer strategies have significant implications for patient access and affordability, raising ongoing concerns regarding the extent to which copay assistance programs can effectively reduce financial barriers to care.

In this study, we examined the impact of CA and CM programs on treatment patterns, medication adherence, and healthcare costs among patients with major depressive disorder (MDD) and bipolar disorder (BPD) who are treated with branded therapies. We chose to focus on MDD and BPD because these chronic mental health conditions are highly prevalent and associated with significant morbidity, functional impairment, and increased healthcare utilization [12,13]. Effective management of these disorders often requires long-term, consistent pharmacologic therapy, and interruptions or gaps in treatment can lead to rapid symptom relapse, worsening of disease severity, and increased risk of hospitalization [14]. We selected often prescribed branded medications for MDD and BPD due to limited or less effective or tolerated generic options [15]. Since branded medications have higher OOP costs, patients rely more on manufacturer copay assistance to maintain access [16]. Even though our analysis is primarily focused on CA and CM programs in the United States, the considerations highlighted, specifically, the shifting of cost burdens to patients, the implementation of complex benefit designs, and the challenges posed by specialty drug costs, are highly relevant beyond the USA in any healthcare jurisdiction that features at least some element of private or mixed funding and where rising healthcare costs are a concern.

We hypothesized that CA and CM programs may have particularly adverse effects in this patient population. Since these programs are designed to prevent manufacturer copay assistance from counting toward a patient’s deductible or OOP maximum, patients can face unexpected and unaffordable medication costs once assistance funds are exhausted. For individuals managing chronic mental health conditions, such financial barriers may lead to reduced medication adherence, treatment discontinuation, and ultimately, poorer clinical outcomes. Furthermore, disruptions in therapy can increase the likelihood of acute episodes requiring costly emergency or inpatient care, thereby raising overall healthcare expenditures.

## 2. Materials and Methods

### 2.1. Data Sources

Kythera Labs’ commercial claims data from January 2020 to December 2024 were used. Kythera data encompass medical and pharmacy claims and represent coverage of 79% of the US patient population [17]. The data set includes both open and closed versions, encompassing approximately 310 million patients, 6.1 million practitioners, 1.6 million organizations, and 1.4 million facilities that collectively generate 40 billion healthcare claims. Kythera contains commercial, Medicare, and Medicaid claims [17]. The open version of the Kythera database comprises claims collected from multiple sources—such as clearinghouses, electronic health records, and pharmacy networks—providing near real-time insights into patient encounters across different insurers and care settings. Open claims offer broad coverage and rapid availability but may lack complete longitudinal detail for each patient. In contrast, the closed version consists of adjudicated claims submitted directly by payers and includes fully reconciled medical and pharmacy transactions for patients continuously enrolled in each insurance plan. While closed claims are smaller in scope and updated less frequently, they ensure complete capture of a patient’s healthcare utilization during the enrollment period, making them particularly useful for outcomes and cost analyses.

We utilized the commercial closed portion which contains data for 172 million enrollees. The data set includes de-identified patient age, sex, ZIP code, diagnoses according to the International Classification of Diseases, Tenth Revision (ICD-10), Current Procedural Terminology codes, and National Drug Codes for medications. Each patient is assigned a unique identifier that links their encounters, allowing for longitudinal analysis. Details of the data have been published elsewhere, and the healthcare outcomes derived from these data have been compared with other data sets for validity and consistency [18].

### 2.2. Study Design and Population

A retrospective cohort design was used. The study period spanned from 1 January 2020 to 31 December 2024. The index date was defined as the first prescription claim for a branded atypical antipsychotic (AAP) or antidepressant (AD) during the identification period, which ran from 1 January 2019 through 31 December 2023. To be included, patients were required to have at least 1 medical claim for a diagnosis of MDD or BPD prior to the index date. If a patient had both BPD and MDD diagnoses, they were classified as having BPD to ensure the MDD and BPD groups were mutually exclusive. Additional inclusion criteria included continuous health plan enrollment for at least 12 months before and after the index date, being at least 18 years of age at the time of the index date and maintaining continuous use of the branded medication for at least 3 months following the index date. Patients who were prescribed both an AAP and an AD were included in both medication groups. The study excluded patients with diagnosis of schizophrenia spectrum or other primary psychotic disorders, and those who received a prescription for a branded AAP (AAP cohort) or a branded AD (AD cohort) in the 12 months prior to the index date.

These exclusion criteria were established to ensure the study population was as homogeneous as possible and to minimize confounding factors. By excluding patients with prior exposure to branded AAPs or ADs in the 1-year pre index date, the study focused on new users or those switching from non-branded alternatives, thereby reducing the risk of carryover effects from previous treatments. Excluding individuals with schizophrenia spectrum or other primary psychotic disorders was essential because these conditions require different treatment approaches and could confound the assessment of outcomes specific to MDD and BPD populations.

Three primary subgroups of patients with MDD or BPD were identified based on their copay arrangements: accumulators (CA), maximizers (CM), and standard copay plans (SCP). CAs are insurance mechanisms that allow patients to use manufacturer copay assistance, but these amounts do not count toward the patient’s deductible or OOP maximum; once the assistance runs out, patients face full cost-sharing until those limits are met. CM spread manufacturer assistance evenly over the year, minimizing monthly OOP medication costs, but like accumulators, payments do not count toward the deductible or OOP maximum. SCPs allow both copay assistance and patient payments to count toward the deductible and OOP maximum, reducing overall OOP medication costs for patients.

Since we used closed claims data from Kythera Labs, patients who moved between different types of healthcare plans during the study patients were excluded. Patients who had a positive copay card use flag were categorized into one of these groups. The CA cohort included patients who had at least 3 prescriptions where their OOP payment exceeded 20% of the drug’s wholesale acquisition cost, surpassed the Affordable Care Act’s mandated annual maximum, showed a decrease in copay card “pay-as-little-as” amounts, or had at least 2 prescriptions with consistently high copay card buydown amounts and minimal reductions in patient costs over time, indicating unsuccessful manufacturer mitigation. The CM cohort consisted of patients with at least 3 prescriptions who maintained the same initial OOP cost for a product within a therapeutic area. The SCP cohort included patients enrolled in traditional coinsurance or copay plans, as well as all other patients who did not meet the criteria for the CA or CM groups.

Patients were categorized by index medication (AAP or AD), diagnosis (MDD or BPD), and copay coverage (CA, CM, or SCP), resulting in 12 analytical cohorts.

### 2.3. Analysis

Outcomes assessed in this study included medication adherence, persistence, discontinuation, treatment abandonment, all-cause and disease-specific pharmacy costs for adherent patients. Adherence was measured using the proportion of days covered, calculated as the ratio of the total days covered by the index medication to the total number of days in the follow-up period. Persistence was defined as the duration of continuous medication use during the follow-up period without a permissible gap in therapy, specifically a gap of ≥60 days for oral medications or ≥30 days for injectable medications. The date of non-persistence was defined as the last day of supply of the final prescription prior to the qualifying gap [19,20]. Persistence was reported as the median number of days from treatment initiation to discontinuation (i.e., the onset of a qualifying gap) during the 12-month follow-up period. Discontinuation was defined by the occurrence of a continuous gap of ≥60 days for oral medications or ≥30 days for injectable medications after the date of next scheduled dose in the days’ supply during the 12-month post-index period, with the discontinuation date marked as the last day of supply from the final filled prescription [19,20]. Patients who experienced at least one such gap were classified as having discontinued therapy. Treatment duration was calculated as the number of days from the initial treatment date to either discontinuation or the date of therapy switch. Treatment abandonment rate was defined as the percentage of patients who reverse (i.e., the prescription was not picked up or paid for) an insurance-approved pharmacy claim for a newly prescribed branded AAP or AD without obtaining a subsequent paid claim for any branded AAP or AD within 90 days [21]. All-cause pharmacy costs for adherent patients were calculated and adjusted to 2024 US dollars using the medical care component of the Consumer Price Index.

Propensity score matching was employed to compare outcomes across health plan designs by index medication within each disease group. The propensity score, defined as the estimated probability of assignment to the case–cohort based on a specific set of covariates, was calculated for all subjects. Each individual in the case–cohort was matched in a 1:1 ratio with a control subject exhibiting the closest propensity score. The matching algorithm incorporated baseline covariates, including patient demographics, socioeconomic status and comorbidities.

The Kythera Labs dataset was utilized to obtain essential demographic variables, including age and sex. Socioeconomic status was assessed using a previously validated composite measure for each US ZIP code, incorporating data on income, education, and occupation derived from the 2021 US Census 5-year estimates [22]. Subjects were subsequently stratified into terciles (low, medium, and high) according to their socioeconomic status summary scores.

To address heterogeneity in patient comorbidities, three established comorbidity indices were employed: the revised Charlson Comorbidity Index (CCI), the Chronic Disease Score (CDS), and the Elixhauser Index. The CCI, originally developed to predict long-term mortality, has been adapted for use with both ICD-9 and ICD-10 codes and has demonstrated reliability and validity across diverse clinical populations [23]. The CDS is a medication-based index that utilizes prescription data to stratify patients according to risk and predict health outcomes [24]. The Elixhauser Index, which classifies comorbidities based on ICD codes, is widely adopted for risk adjustment in health services research [25]. Incorporating these indices into the matching algorithm has been shown to enhance the robustness of resulting estimators [26]. Additional adjustments were made for both mental and systemic comorbidities.

Descriptive analyses were conducted for all covariates. Categorical variables were summarized using frequencies and percentages, whereas continuous variables were described by means and standard deviations. Statistical comparisons between cohorts were performed using *t*-tests for continuous variables and Pearson’s chi-square tests for categorical variables, with statistical significance defined as *p* < 0.05. Standardized differences were also calculated for each variable. Following propensity score matching, it was expected that no significant differences would remain between the two patient cohorts with respect to pre-index characteristics; therefore, all associated differences in outcomes were linked to health benefit design. All analyses were performed using PySpark 4.0 and SparkR 4.0 within the Databricks and R environments.

## 3. Results

After applying the inclusion and exclusion criteria, we identified 263,746 patients. This population included 53,590 patients with MDD treated with branded AAPs, 81,472 with BPD treated with branded AAPs, 112,670 with MDD treated with branded ADs, and 16,014 with BPD treated with branded ADs (Supplementary Table S1).

Of the total cohort, 50,293 patients met the additional criteria of having a positive copay card use flag and maintaining continuous use of branded medication for at least 3 months post-index. Copay plan distribution is presented in Table 1.

**Figure 1:**
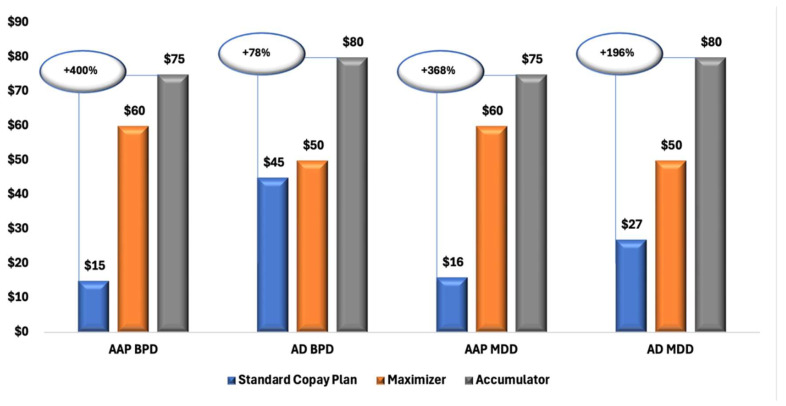
Median Copay/Coinsurance Amounts for Branded AAP and AD Prescriptions Among Patients with MDD and BPD, by Copay Plan Type. Abbreviations: AAP, atypical antipsychotics; AD, antidepressants; BPD, bipolar disorder; MDD, major depressive disorder; SCP, standard copay plan.

Figure 1 illustrates a clear trend of increasing OOP costs for patients enrolled in CA and CM plans compared with those in SCPs. Across all diagnostic and treatment groups, both MDD and BPD, and branded AAPs and ADs, patients in CA plans consistently experienced the highest financial burden, followed by those in CM plans.

### Descriptive Analysis

Supplemental Table S2 presents the baseline characteristics of MDD by different insurance design for branded AAPs and branded APs.

Among patients with MDD treated with branded AAPs, those in CA and CM plans were significantly younger than those in SCPs, with mean ages of 40.03 and 40.94 vs. 43.20 years (both *p* < 0.0001). Distribution by patient sex was similar across cohorts (72.95–75.57% female). Geographic variation showed that CA patients had lower representation in the Northeast and South regions and higher representations in the Midwest (*p* < 0.0001); fewer CM patients resided in the West and more in the Midwest (*p* < 0.01). Across all comorbidity indices (CCI, Elixhauser Index, and CDS), CA and CM patients had significantly lower scores than those in SCPs (*p* < 0.0001).

Among patients with MDD treated with branded ADs, those in CA and CM plans were also younger than those in SCPs, with mean ages of 40.71 and 41.68 years vs. 44.58 years (*p* < 0.0001). The CA group had a higher proportion of females (74.72% vs. 71.29%, *p* = 0.0159). Patients in both the CA and CM groups were more likely to reside in the Midwest and less likely to reside in the South US region than those in SCPs. Consistent with findings for branded AAPs, patients in the CA and CM groups for branded ADs had lower comorbidity indices than those in SCPs.

Supplemental Table S3 summarizes the baseline characteristics of patients with BPD by insurance type for both branded AAPs and branded ADs. The findings closely mirror those observed in the MDD group: patients in CA and CM plans were significantly younger than those in standard commercial plans for both branded AAPs (mean ages: 37.13 and 36.91 years vs. 40.06 years; *p* < 0.01) and branded ADs (39.25 vs. 44.56 years; *p* < 0.01). Distribution by patient sex showed a higher proportion of males in both CAP groups. Similarly to MDD, patients with BPD in the CA and CM cohorts had lower comorbidity scores than those in standard commercial plans (Supplemental Table S3).

**Figure 2:**
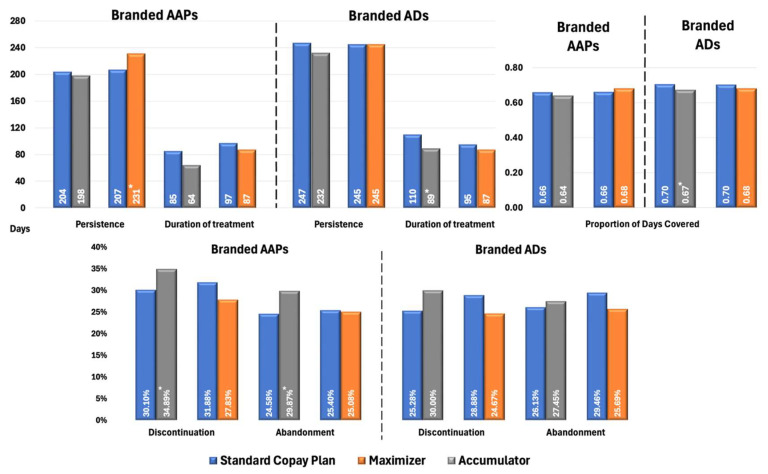
Propensity Score-Matched Treatment Patterns for Patients with MDD Treated with Branded AAPs and/or Branded ADs. * Indicates statistically significant difference (*p* < 0.05) compared with SCPs Abbreviations: AAP, atypical antipsychotics; AD, antidepressants; MDD, major depressive disorder; SCP, standard copay plan.

Figure 2 presents propensity score-adjusted differences in Proportion of Days Covered (PDC), persistence, treatment duration, discontinuation, and treatment abandonment rates between branded AAP and AD groups for patients with MDD. Patients in CA plans using branded AAPs exhibited marginally lower adherence (PDC: 0.64 vs. 0.66; *p* = 0.05) and significantly higher rates of discontinuation (34.89% vs. 30.10%, *p* < 0.02) and treatment abandonment (29.87% vs. 24.58%, *p* < 0.05) than those in SCPs. Patients in CM plans had significantly longer treatment persistence compared with SCPs (231 vs. 207 days; *p* < 0.05); however, other metrics were similar between the two cohorts, with no significant differences observed. This alignment likely stems from CM plans’ payment structure, which smooths OOP costs over time, unlike CA plans, resulting in minimal impact on treatment continuity.

In terms of branded ADs, the figure shows CA had lower adherence (PDC: 0.67 vs. 0.70; *p* < 0.01) and shorter treatment duration (89 vs. 110 days; *p* < 0.03) than SCPs, with no significant differences in discontinuation and treatment abandonment rates compared with significant differences observed in AAPs. Branded ADs generally have lower OOP costs than AAPs, making patients less sensitive to the financial impact when copay assistance is exhausted under CA plans.

**Figure 3:**
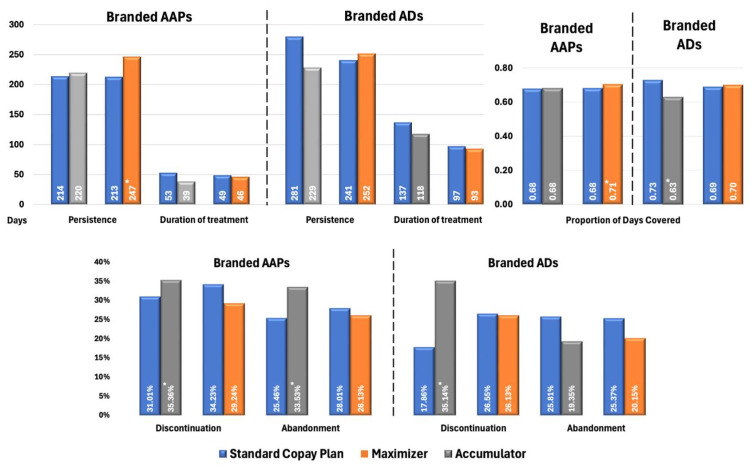
Propensity Score-Matched Treatment Patterns for Patients with BPD Treated with Branded AAPs and/or Branded ADs. * Indicates statistically significant difference (*p* < 0.05) compared with SCPs Abbreviations: AAP, atypical antipsychotics; AD, antidepressants; BPD, bipolar disorder; SCP, standard copay plan.

Figure 3 illustrates the impact of health benefit designs on treatment patterns in patients with BPD. For branded AAPs, patients enrolled in CA programs demonstrated significantly higher rates of discontinuation (35.36% vs. 31.01%, *p* < 0.02) and treatment abandonment (33.53% vs. 25.46%, *p* < 0.01) compared with SCPs. Similarly to patterns observed in MDD cohorts, the CM had higher adherence (PDC: 0.71 vs. 0.68; *p* < 0.03) and longer persistence (247 vs. 213 days; *p* < 0.01) while other metrics were not significantly different.

Among patients with BPD using branded ADs, those in CA plans demonstrated significantly poorer adherence (PDC: 0.63 vs. 0.73; *p* < 0.02) and higher discontinuation rates (35.14% vs. 17.86%, *p* < 0.02) compared with SCPs. However, no significant differences were observed in treatment abandonment rates. When copay assistance is exhausted, patients face sudden OOP costs, which may result in them intermittently skipping doses or delaying refills [8,9] (captured as discontinuation) without exceeding the ≥60-day gap threshold that defines abandonment. Crucially, ADs are often prescribed alongside mood stabilizers and antipsychotics in BPD, leading patients to deprioritize them first during financial strain while maintaining core therapies. This results in fragmented AD use (elevating discontinuation) without complete therapy termination (hence unchanged abandonment). Similarly to the MDD population, no significant differences in treatment patterns were observed between patients with BPD using ADs under CM plans and those under SCPs.

**Figure 4:**
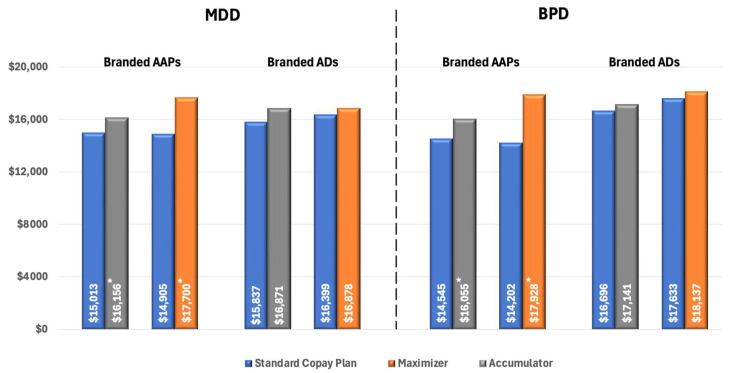
All-cause Pharmacy Costs for Patients with MPR ≥ 0.80 (PPPY). * Indicates statistically significant difference (*p* < 0.05) compared with SCPs. Abbreviations: AAP, atypical antipsychotics; AD, antidepressants; BPD, bipolar disorder; MDD, major depressive disorder.

To eliminate the confounding effect of non-adherence, we compared pharmacy costs across different insurance designs in patients who were adherent to treatment. Among these patients with MDD using AAPs, pharmacy costs were significantly higher for those enrolled in CA plans ($16,156 vs. $15,013 for SCPs, *p* < 0.02) and CM plans ($17,699 vs. $14,904 for SCPs, *p* < 0.01). There was no significant difference in pharmacy costs between insurance designs among patients with branded ADs (Figure 4).

For patients with BPD using branded AAPs, we observed a similar pattern of increased pharmacy costs associated with CA and CM programs. Specifically, among adherent patients, those enrolled in CA plans had significantly higher pharmacy costs compared to those in SCPs ($16,055 vs. $14,545, *p* < 0.01), and cost differences were even higher for patients in CM plans ($17,928 vs. $14,202, *p* < 0.01) (Figure 4).

## 4. Discussion

Our findings add to the growing body of evidence showing that cost-shifting programs can negatively impact patient care and the overall health system [11,27,28,29]. These programs are associated with reduced medication adherence and increased financial burdens for patients. In our study, both the CA and CM cohorts faced significantly higher median OOP costs compared to those on SCPs, with CA patients incurring costs that were up to 4 times higher than those on SCPs.

Recent research showed a significant deficiency in payer transparency concerning CA and CM programs [11]. Patients are frequently uninformed about their enrollment in these programs, which can result in unexpected financial liabilities when copay assistance resources are exhausted [28]. Unexpected OOP costs have been shown to significantly affect medication adherence and persistence among patients with chronic mental health conditions [30]. In our analysis, patients participating in cost accumulator programs demonstrated lower adherence to prescribed therapies and higher rates of treatment discontinuation in both MDD and BPD cohorts. These findings are consistent with results from studies in other therapeutic areas. Research examining patients with autoimmune diseases found that CA programs were linked to decreased adherence and persistence with specialty medications [27]. Another study showed higher medication adherence and persistence in states that banned CA programs compared with states that allowed such programs [31]. RxCrossroads by McKesson’s data and research showed that implementation of cost accumulator programs was associated with a 1.5-fold decrease in prescription fill rates and a 13% reduction in treatment persistence following the exhaustion of benefit caps [32]. The consequences of non-adherence are substantial, with estimates suggesting that inadequate medication adherence may cost the United States healthcare system up to $528 billion annually, primarily due to accelerated disease progression and increased utilization of costly healthcare services resulting from premature treatment discontinuation [33].

More than half of Americans depend on employer-sponsored health insurance, that average premium increased to $8951 for individual coverage and $25,572 for family coverage, an annual increase of 6% and 7%, respectively, outpacing both wage growth and inflation [34]. Therefore, there is a growing employer interest in cost-containment strategies such as CA and CM programs. By 2024, 42% of self-insured employers had adopted either CA or CM programs, with an additional 8% indicating plans to implement or consider these programs within the subsequent 2 years [35]. Despite this widespread adoption, fewer than half of employers reported these programs to be highly effective in controlling healthcare expenditures [35]. Consistent with these perceptions, our analysis demonstrates that both CA and CM programs are associated with increased pharmacy costs among adherent patients, underscoring concerns regarding the efficacy of these interventions as cost-management tools.

Concerns about the negative impact of these programs on patient care have led to legal challenges and new regulations aimed at limiting patients’ OOP costs [10]. At the state level, progress has been made: 21 states, along with Washington, DC, and Puerto Rico, have enacted laws requiring insurers to apply third-party payments toward patients’ annual cost-sharing obligations [36]. These laws primarily target CA programs, though some also address CM programs. While such legislation has helped improve patient access to medications and treatment adherence, gaps remain since not all types of health plans are covered—highlighting a need for possible federal intervention. On the federal front, regulatory changes and ongoing litigation have introduced uncertainty. Recent Centers for Medicare & Medicaid Services rules have clarified protections for prescription drugs, but further action on CA programs has been postponed, suggesting that future policy making could have a substantial impact on cost-sharing policies for a broader range of health plans [37]. Despite concerns about their negative consequences, CA and CM programs remain widely used, with over 40% of working adults currently covered by health plans that employ these cost-shifting initiatives, and payers are expected to expand them even further [35]. In light of this trend, our paper aims to shed light on both the intended and unintended effects of these increasingly common programs, specifically for patients with MDD and BPD. Future research should prioritize several key areas to deepen our understanding of copay assistance programs’ dynamics. First, investigations into how health plan types (e.g., high-deductible vs. traditional plans) influence prescribing decisions are warranted, particularly given evidence that plan structures directly impact medication adherence and financial burden. Second, comprehensive survey studies assessing patient and prescriber awareness of copay assistance programs, including CA, CM, and SCP, could elucidate knowledge gaps and attitudinal barriers, building on existing research highlighting concerns about program sustainability and transparency. Third, risk-adjusted analysis comparing CA, CM, and SCP interventions would provide critical insights into whether these programs inadvertently create barriers to branded medication adoption. Such a study could examine switches from generic to branded therapies and address unanswered questions about plan-level incentives and prescriber behavior, ultimately informing more equitable access policies.

Collectively, these directions would bridge gaps in program efficacy, stakeholder education, and systemic incentives, advancing toward optimized patient-centered solutions.

### Limitations

Our study has several limitations typical of observational research. First, although Kythera Labs commercial claims data represent approximately 79% of the US population and include over 170 million enrollees, they may still contain omissions or misclassifications of diagnoses and treatments. However, this limitation was mitigated by using the closed claims dataset to enhance completeness and by applying validated coding algorithms to improve accuracy. Second, the study included only adults with MDD or BPD covered by commercial insurance in the United States, which may limit generalizability to publicly insured or uninsured populations. Finally, the longitudinal design required patients to initiate branded medication use between 2021 and 2023, with follow-up spanning from January 2020 through December 2024. Although this design captures emerging trends in copay accumulator and maximizer programs, the relatively short post-index follow-up period (12 months) and the requirement for at least three months of continuous branded medication use may limit observation of long-term adherence, persistence, and health outcomes. 

## 5. Conclusions

Patients with MDD and BPD enrolled in CA and CM programs faced significantly higher OOP costs. Those in CA programs, in particular, experienced poorer medication adherence, higher rates of treatment abandonment and pharmacy costs. These findings highlight the need for policymakers to carefully consider the effects of such benefit structures when designing coverage for vulnerable mental health populations.

## Figures and Tables

**Table 1:** Number of Study Participants by Copay Plan Type Among Patients with Positive Copay Card Use and ≥3-Month Continuous Branded Medication Use.

Health Plan Type	Patients with Branded AAP Use		Patients with Branded AD Use	
	MDD	BPD	MDD	BPD
Standard Copay Plan	8997	11,957	20,731	2295
Copay Accumulator Plan	1135	1363	1060	93
Copay Maximizer Plan	618	1221	689	134
Total (MDD/BPD)	10,750	14,541	22,480	2522
Total (AP/AD)	25,291		25,002	
Grand total	50,293			

Abbreviations: AAP: atypical antipsychotic; AD; antidepressant; BPD: bipolar disorder; MDD: major depressive disorder.

## Data Availability

The data that support the findings of this study are available from Kythera Labs. Due to restrictions on the availability of these data, which were used under license for the current study, they are not publicly available. However, data may be made available from the corresponding author, O. Baser, upon reasonable request and with permission of Kythera Labs.

## Supplementary Materials

The following supporting information can be downloaded at: https://www.mdpi.com/article/10.3390/jmahp13040055/s1, Table S1: Patient Attrition by Diagnosis and Branded Medication Type; Table S2: Baseline Characteristics for Patients with MDD by Treatment Cohort; Table S3: Baseline Characteristics: Patients with Bipolar Disorder by Treatment Cohort.

## Abbreviations

The following abbreviations are used in this manuscript:

AD	Antidepressant
AAP	Atypical antipsychotic
BPD	Bipolar disorder
CA	Copay accumulator
CCI	Charlson Comorbidity Index
CDS	Chronic Disease Score
CM	Copay maximizer
ICD-10	International Classification of Diseases, Tenth Revision
MDD	Major depressive disorder
OOP	Out-of-pocket
SCP	Standard copay plan
