# Supplementary material for: The Impact of Copay Accumulators and Maximizers on Treatment Patterns, Adherence, and Costs Among Patients with Major Depressive and Bipolar Disorders Treated with Branded Therapies
Source: J Mark Access Health Policy. 2025 Nov 7;13(4):55. doi: 10.3390/jmahp13040055 (PMC12641712; doi:10.3390/jmahp13040055)
Supplement: Supplementary file 1 [file jmahp-13-00055-s001.zip › jmahp-3822843-supplementary.pdf]

Supplementary Table S1. Patient Attrition by Diagnosis and Branded Medication Type.

Inclusion Criteria		Branded AAP		Branded AD	
		MDD	BPD	MDD	BPD
a)	≥1 pharmacy claim for an AP/AD in the identification period (01/01/21–12/31/23; the first AP/AD initiation date during the identification period = index date)	172,897	321,184	284,861	49,257
b)	≥1 medical claim for MDD/BP diagnosis before the index date; if a patient had both BPD and MDD diagnoses, they were classified as BPD	110,341	203,145	175,717	27,998
c)	≥18 years of age on the index date	100,931	197,685	171,806	27,697
d)	Continuous health plan enrollment for 12 months before and after index date	72,088	135,420	139,805	20,561
**Exclusion Criteria**					
a)	≥1 diagnosed with schizophrenia spectrum and other primary psychotic disorders (F20-F29) in the 12 months pre index date	62,248	104,572	137,623	18,709
b)	≥1 brand AP/AD usage in the 12 months pre index date	53,590	81,472	112,670	16,014
	Total	135,062		128,684	

AAP: atypical antipsychotic; AD: antidepressant; BPD: bipolar disorder; MDD: major depressive disorder.

Supplementary Table S2. Baseline Characteristics for Patients with MDD by Treatment Cohort

	Patients with MDD																							
	**Branded AAPs**												**Branded ADs**											
	**Copay Accumulators vs Standard Copay Plans**						**Copay Maximizers vs Standard Copay Plans**						**Copay Accumulators vs Standard Copay Plans**						**Copay Maximizers vs Standard Copay Plans**					
	**Copay Accumulators (N = 1,135)**		**Standard Copay Plans (N = 8,997)**		**P-value**	**Std. diff**	**Copay Maximizers (N = 618)**		**Standard Copay Plans (N = 8,997)**		**P-value**	**Std. diff**	**Copay Accumulators (N = 1,060)**		**Standard Copay Plans (N = 20,731)**		**P-value**	**Std. diff**	**Copay Maximizers (N = 689)**		**Standard Copay Plans (N = 20,731)**		**P-value**	**Std. diff**
**Age**	**Mean**	**SD**	**Mean**	**SD**			**Mean**	**SD**	**Mean**	**SD**			**Mean**	**SD**	**Mean**	**SD**			**Mean**	**SD**	**Mean**	**SD**		
Mean Age	40.03	13.35	43.2	15.41	<.0001	0.2088	40.94	13.65	43.2	15.41	<.0001	0.1479	40.71	12.56	44.58	14.34	<.0001	0.2715	41.68	12.83	44.58	14.34	<.0001	0.2032
**Age Groups**	**N**	**%**	**N**	**%**			**N**	**%**	**N**	**%**			**N**	**%**	**N**	**%**			**N**	**%**	**N**	**%**		
Age 18-34 years	442	38.94%	2,964	32.94%	<.0001	0.1271	221	35.76%	2,964	32.94%	0.1502	0.0598	364	34.34%	5,712	27.55%	<.0001	0.1514	211	30.62%	5,712	27.55%	0.0762	0.0687
Age 35-	272	23.9	1,8	20.1	0.0	0.0	128	20.7	1,8	20.1	0.7	0.0	274	25.8	4,8	23.4	0.0	0.0	189	27.4	4,8	23.4	0.0	0.0

	Patients with MDD																							
	Branded AAPs												Branded ADs											
	Copay Accumulators vs Standard Copay Plans						Copay Maximizers vs Standard Copay Plans						Copay Accumulators vs Standard Copay Plans						Copay Maximizers vs Standard Copay Plans					
	Copay Accumulators (N = 1,135)		Standard Copay Plans (N = 8,997)		P-value	Std. diff	Copay Maximizers (N = 618)		Standard Copay Plans (N = 8,997)		P-value	Std. diff	Copay Accumulators (N = 1,060)		Standard Copay Plans (N = 20,731)		P-value	Std. diff	Copay Maximizers (N = 689)		Standard Copay Plans (N = 20,731)		P-value	Std. diff
44 years		6%	13	5%	027	944		1%	13	5%	369	14		5%	70	9%	779	555		3%	70	9%	166	928
Age ≥45	421	37.09%	4,220	46.90%	<.001	0.1973	269	43.53%	4,220	46.90%	0.1036	0.0677	422	39.81%	10,149	48.96%	<.001	0.1831	289	41.94%	10,149	48.96%	0.0003	0.1403
**Sex**	**N**	**%**	**N**	**%**			**N**	**%**	**N**	**%**			**N**	**%**	**N**	**%**			**N**	**%**	**N**	**%**		
Female	845	74.45%	6,563	72.95%	0.2819	0.0339	467	75.57%	6,563	72.95%	0.1553	0.0591	792	74.72%	14,779	71.29%	0.0159	0.0759	481	69.81%	14,779	71.29%	0.3991	0.0327
Male	290	25.55%	2,433	27.04%	0.2854	0.0336	151	24.43%	2,433	27.04%	0.157	0.0588	268	25.28%	5,952	28.71%	0.0159	0.0759	208	30.19%	5,952	28.71%	0.3991	0.0327
**Index Year**	**N**	**%**	**N**	**%**			**N**	**%**	**N**	**%**			**N**	**%**	**N**	**%**			**N**	**%**	**N**	**%**		
2021	230	20.26%	2,584	28.72%	<.001	0.1891	147	23.79%	2,584	28.72%	0.0085	0.1094	597	56.32%	7,652	36.91%	<.001	0.4017	248	35.99%	7,652	36.91%	0.6237	0.019
2022	320	28.19%	3,180	35.35%	<.001	0.1505	181	29.29%	3,180	35.35%	0.0023	0.1271	220	20.75%	6,611	31.89%	<.001	0.2403	126	18.29%	6,611	31.89%	<.001	0.2933
2023	585	51.54%	3,233	35.93%	<.001	0.3237	290	46.93%	3,233	35.93%	<.001	0.2285	243	22.92%	6,468	31.20%	<.001	0.1794	315	45.72%	6,468	31.20%	<.001	0.3126
**US Geographic Region**	**N**	**%**	**N**	**%**			**N**	**%**	**N**	**%**			**N**	**%**	**N**	**%**			**N**	**%**	**N**	**%**		
Northeast	117	10.31%	1,475	16.39%	<.001	0.1675	101	16.34%	1,475	16.39%	0.9734	0.0014	130	12.26%	2,927	14.12%	0.0899	0.0534	110	15.97%	2,927	14.12%	0.1717	0.0529
Midwest	419	36.92%	1,906	21.18%	<.001	0.3767	165	26.70%	1,906	21.18%	0.0013	0.1342	363	34.25%	4,508	21.75%	<.001	0.3007	198	28.74%	4,508	21.75%	<.001	0.1689
South	415	36.56%	3,906	43.41%	<.001	0.1386	246	39.81%	3,906	43.41%	0.0798	0.0729	396	37.36%	9,360	45.15%	<.001	0.1568	251	36.43%	9,360	45.15%	<.001	0.1754
West	133	11.72%	1,358	15.09%	0.0025	0.0953	68	11.00%	1,358	15.09%	0.0056	0.1151	118	11.13%	3,004	14.49%	0.0023	0.0959	86	12.48%	3,004	14.49%	0.1399	0.0572
**Socioeconomic Status**	**N**	**%**	**N**	**%**			**N**	**%**	**N**	**%**			**N**	**%**	**N**	**%**			**N**	**%**	**N**	**%**		
Low tercile	292	25.73%	3,009	33.44%	<.001	0.1649	171	27.67%	3,009	33.44%	0.0032	0.1228	322	30.38%	6,780	32.70%	0.1149	0.0497	196	28.45%	6,780	32.70%	0.019	0.0909
Middle tercile	395	34.80%	2,892	32.14%	0.0715	0.0568	208	33.66%	2,892	32.14%	0.4363	0.0324	386	36.42%	6,685	32.25%	0.0047	0.0891	236	34.25%	6,685	32.25%	0.268	0.0429
High tercile	432	38.06%	2,919	32.44%	0.0002	0.1195	229	37.06%	2,919	32.44%	0.0181	0.0983	342	32.26%	6,919	33.38%	0.4542	0.0236	248	35.99%	6,919	33.38%	0.1518	0.0555
**Comorbidity Index Scores**	**Mean**	**SD**	**Mean**	**SD**			**Mean**	**SD**	**Mean**	**SD**			**Mean**	**SD**	**Mean**	**SD**			**Mean**	**SD**	**Mean**	**SD**		
Charlson Comorbidity Index	0.33	0.76	0.59	1.1	<.001	0.2463	0.31	0.75	0.59	1.1	<.001	0.2593	0.28	0.65	0.47	0.97	<.001	0.1961	0.3	0.71	0.47	0.97	<.001	0.1748

	Patients with MDD																							
	Branded AAPs												Branded ADs											
	Copay Accumulators vs Standard Copay Plans						Copay Maximizers vs Standard Copay Plans						Copay Accumulators vs Standard Copay Plans						Copay Maximizers vs Standard Copay Plans					
	Copay Accumulators (N = 1,135)		Standard Copay Plans (N = 8,997)		P-value	Std. diff	Copay Maximizers (N = 618)		Standard Copay Plans (N = 8,997)		P-value	Std. diff	Copay Accumulators (N = 1,060)		Standard Copay Plans (N = 20,731)		P-value	Std. diff	Copay Maximizers (N = 689)		Standard Copay Plans (N = 20,731)		P-value	Std. diff
score																								
Elixhauser Index score	2.14	1.65	2.72	2.18	<.0001	0.2708	2.16	1.62	2.72	2.18	<.0001	0.2588	2	1.46	2.45	2	<.0001	0.2254	2.08	1.6	2.45	2	<.0001	0.1871
Chronic Disease Score	4.2	2.99	4.55	3.32	0.0002	0.1067	4.25	2.86	4.55	3.32	0.0012	0.0919	3.78	2.86	3.68	3.15	0.2568	0.0327	3.73	2.97	3.68	3.15	0.6608	0.0161
**Mental Health Comorbidities**	N	%	N	%			N	%	N	%			N	%	N	%			N	%	N	%		
Depression	466	41.06%	4,151	46.14%	0.0012	0.1021	270	43.69%	4,151	46.14%	0.2375	0.0491	429	40.47%	9,172	44.24%	0.0159	0.076	279	40.49%	9,172	44.24%	0.0512	0.0755
Generalized anxiety disorder	532	46.87%	3,742	41.59%	0.0007	0.107	309	50.00%	3,742	41.59%	<.0001	0.1704	480	45.28%	8,780	42.35%	0.0597	0.0593	337	48.91%	8,780	42.35%	0.0006	0.1327
Obsessive-compulsive disorder	56	4.93%	367	4.08%	0.1749	0.0427	30	4.85%	367	4.08%	0.3488	0.039	36	3.40%	679	3.28%	0.8293	0.0068	36	5.22%	679	3.28%	0.0051	0.1086
Panic disorder	92	8.11%	676	7.51%	0.4776	0.0224	52	8.41%	676	7.51%	0.413	0.034	80	7.55%	1,496	7.22%	0.685	0.0128	73	10.60%	1,496	7.22%	0.0008	0.1297
Substance use disorders	178	15.68%	2,148	23.87%	<.0001	0.1951	108	17.48%	2,148	23.87%	0.0003	0.1511	103	9.72%	2,989	14.42%	<.0001	0.1348	86	12.48%	2,989	14.42%	0.1539	0.0552
Post-traumatic stress disorder	140	12.33%	1,382	15.36%	0.0072	0.0847	97	15.70%	1,382	15.36%	0.8232	0.0093	114	10.75%	2,374	11.45%	0.4866	0.0219	84	12.19%	2,374	11.45%	0.5487	0.0232
**Systemic Health Comorbidities**	N	%	N	%			N	%	N	%			N	%	N	%			N	%	N	%		
Obesity	192	16.92%	1,745	19.40%	0.0453	0.063	122	19.74%	1,745	19.40%	0.8335	0.0087	168	15.85%	3,792	18.29%	0.0443	0.0633	128	18.58%	3,792	18.29%	0.8484	0.0074
Diabetes	25	2.20%	547	6.08%	<.0001	0.1682	19	3.07%	547	6.08%	0.0021	0.1277	10	0.94%	578	2.79%	0.0003	0.1139	14	2.03%	578	2.79%	0.2336	0.0461
Viral hepatitis	4	0.35%	112	1.24%	0.0077	0.0839	0	0.00%	112	1.24%	0.0053	0.1161	4	0.38%	91	0.44%	0.7665	0.0093	1	0.15%	91	0.44%	0.246	0.0449
Constipation	64	5.64%	538	5.98%	0.647	0.0144	30	4.85%	538	5.98%	0.251	0.0477	36	3.40%	1,161	5.60%	0.0021	0.0968	28	4.06%	1,161	5.60%	0.0831	0.0671
Parkinson's	0	0.00%	86	0.96%	0.0009	0.1042	1	0.16%	86	0.96%	0.0437	0.0839	2	0.19%	70	0.34%	0.4097	0.026	1	0.15%	70	0.34%	0.3871	0.0335

	Patients with MDD																							
	**Branded AAPs**												**Branded ADs**											
	**Copay Accumulators vs Standard Copay Plans**						**Copay Maximizers vs Standard Copay Plans**						**Copay Accumulators vs Standard Copay Plans**						**Copay Maximizers vs Standard Copay Plans**					
	**Copay Accumulators (N = 1,135)**		**Standard Copay Plans (N = 8,997)**		**P-value**	**Std. diff**	**Copay Maximizers (N = 618)**		**Standard Copay Plans (N = 8,997)**		**P-value**	**Std. diff**	**Copay Accumulators (N = 1,060)**		**Standard Copay Plans (N = 20,731)**		**P-value**	**Std. diff**	**Copay Maximizers (N = 689)**		**Standard Copay Plans (N = 20,731)**		**P-value**	**Std. diff**
disease																								
Chronic obstructive pulmonary disease	16	1.41%	485	5.39%	<.0001	0.1839	6	0.97%	485	5.39%	<.0001	0.201	12	1.13%	639	3.08%	0.0003	0.1146	8	1.16%	639	3.08%	0.0037	0.1123
Chronic pain	76	6.70%	880	9.78%	0.0008	0.1056	35	5.66%	880	9.78%	0.0007	0.1404	66	6.23%	1,554	7.50%	0.1243	0.0484	36	5.22%	1,554	7.50%	0.0253	0.0866
Cancer	1	0.09%	0	0.00%	0.0049	0.0887	0	0.00%	0	0.00%	<.0001		0	0.00%	7	0.03%	0.5496	0.0188	0	0.00%	7	0.03%	0.6295	0.0187
Atrial fibrillation	2	0.18%	142	1.58%	0.0002	0.1185	2	0.32%	142	1.58%	0.013	0.1033	6	0.57%	353	1.70%	0.0046	0.0893	6	0.87%	353	1.70%	0.0942	0.0648
Cardiovascular disease	19	1.67%	298	3.31%	0.0028	0.0941	10	1.62%	298	3.31%	0.0207	0.0962	5	0.47%	596	2.87%	<.0001	0.1468	8	1.16%	596	2.87%	0.0075	0.1035
Peripheral vascular disease	7	0.62%	139	1.54%	0.0134	0.0779	2	0.32%	139	1.54%	0.0146	0.1016	2	0.19%	221	1.07%	0.0056	0.0872	1	0.15%	221	1.07%	0.0189	0.0909

Abbreviations: AAP, atypical antipsychotics; AD, antidepressant; MDD, major depressive disorder; SD, standard deviation; Std. diff., standardized difference.

Supplementary Table S3. Baseline Characteristics: Patients with Bipolar Disorder by Treatment Cohort**.**

	Patients with BPD																							
	Branded AAPs												Branded ADs											
	Copay Accumulators vs Standard Copay Plans						Copay Maximizers vs Standard Copay Plans						Copay Accumulators vs Standard Copay Plans						Copay Maximizers vs Standard Copay Plans					
	Copay Accumulators (N = 1,363)		Standard Copay Plans (N = 11,957)		P-value	Std. diff.	Copay Maximizers (N = 1,221)		Standard Copay Plans (N = 11,957)		P-value	Std. diff.	Copay Accumulators (N = 93)		Standard Copay Plans (N = 2,295)		P-value	Std. diff.	Copay Maximizers (N = 134)		Standard Copay Plans (N = 2,295)		P-value	Std. diff.
**Age**	Mean	SD	Mean	SD			Mean	SD	Mean	SD			Mean	SD	Mean	SD			Mean	SD	Mean	SD		
Mean Age	37.13	12.2	**40.06**	13.44	<.0001	0.2203	36.91	11.96	**40.06**	13.44	<.0001	0.2367	39.25	12.2	**44.56**	14.05	0.0003	0.3801	42.61	11.88	44.56	14.05	0.0698	0.1398
**Age Groups**	N	%	N	%			N	%	N	%			N	%	N	%			N	%	N	%		
18-34 years	638	**46.81%**	4,709	39.38%	<.0001	0.1516	579	**47.42%**	4,709	39.38%	<.0001	0.1641	40	**43.01%**	635	27.67%	0.0013	0.3413	34	25.37%	635	27.67%	0.5631	0.0514
35-44 years	329	24.14%	2,813	23.53%	0.6141	0.0144	298	24.41%	2,813	23.53%	0.4902	0.0207	18	19.35%	532	23.18%	0.3903	0.0908	39	29.10%	532	23.18%	0.116	0.1397
≥45 years	396	29.05%	4,435	**37.09%**	<.0001	0.1674	344	28.17%	4,435	**37.09%**	<.0001	0.1857	35	37.63%	1,128	**49.15%**	0.0294	0.2305	61	45.52%	1,128	49.15%	0.4142	0.0726
**Sex**	N	%	N	%			N	%	N	%			N	%	N	%			N	%	N	%		
Female	988	72.49%	8,775	73.39%	0.4763	0.0204	860	70.43%	8,775	**73.39%**	0.0266	0.0666	54	58.06%	1,685	**73.42%**	0.0011	0.3458	103	76.87%	1,685	73.42%	0.3791	0.0781
Male	375	27.51%	3,182	26.61%	0.4763	0.0204	361	**29.57%**	3,182	26.61%	0.0266	0.0666	39	**41.94%**	610	26.58%	0.0011	0.3458	31	23.13%	610	26.58%	0.3791	0.0781
**Index Year**	N	%	N	%			N	%	N	%			N	%	N	%			N	%	N	%		
2021	373	27.37%	4,284	**35.83%**	<.0001	0.1777	318	26.04%	4,284	**35.83%**	<.0001	0.2056	47	**50.54%**	777	33.86%	0.0009	0.3516	32	23.88%	777	**33.86%**	0.0172	0.2118
2022	459	33.68%	4,857	**40.62%**	<.0001	0.1419	422	34.56%	4,857	**40.62%**	<.0001	0.1237	22	23.66%	739	32.20%	0.083	0.1834	29	21.64%	739	**32.20%**	0.0106	0.2273
2023	531	**38.96%**	2,816	23.55%	<.0001	0.3573	481	**39.39%**	2,816	23.55%	<.0001	0.3678	24	25.81%	779	33.94%	0.1035	0.1723	73	**54.48%**	779	33.94%	<.0001	0.4322
**US Geographic Region**	N	%	N	%			N	%	N	%			N	%	N	%			N	%	N	%		
Northeast	161	11.81%	2,174	**18.18%**	<.0001	0.1677	172	14.09%	2,174	**18.18%**	0.0004	0.1071	10	10.75%	386	16.82%	0.1231	0.1631	15	11.19%	386	16.82%	0.0882	0.1515
Midwest	440	**32.28%**	2,258	18.88%	<.0001	0.335	341	**27.93%**	2,258	18.88%	<.0001	0.2278	24	25.81%	456	19.87%	0.1613	0.1481	29	21.64%	456	19.87%	0.6178	0.0443
South	495	36.32%	5,156	**43.12%**	<.0001	0.1378	520	42.59%	5,156	43.12%	0.7201	0.0108	41	44.09%	1,038	45.23%	0.8281	0.023	66	49.25%	1,038	45.23%	0.3631	0.0808
West	206	15.11%	1,995	16.68%	0.139	0.0423	126	10.32%	1,995	**16.68%**	<.0001	0.1734	13	13.98%	313	13.64%	0.9254	0.0099	12	8.96%	313	13.64%	0.1217	0.1376
**Socioeconomic Status**	N	%	N	%			N	%	N	%			N	%	N	%			N	%	N	%		
Low tercile	357	26.19%	4,008	**33.52%**	<.0001	0.1563	351	28.75%	4,008	**33.52%**	0.0007	0.1015	23	24.73%	759	33.07%	0.0929	0.1778	37	27.61%	759	33.07%	0.1906	0.1163

	Patients with BPD																							
	Branded AAPs												Branded ADs											
	Copay Accumulators vs Standard Copay Plans						Copay Maximizers vs Standard Copay Plans						Copay Accumulators vs Standard Copay Plans						Copay Maximizers vs Standard Copay Plans					
	Copay Accumulators (N = 1,363)		Standard Copay Plans (N = 11,957)		P-value	Std. diff.	Copay Maximizers (N = 1,221)		Standard Copay Plans (N = 11,957)		P-value	Std. diff.	Copay Accumulators (N = 93)		Standard Copay Plans (N = 2,295)		P-value	Std. diff.	Copay Maximizers (N = 134)		Standard Copay Plans (N = 2,295)		P-value	Std. diff.
Middle tercile	505	**37.05%**	3,801	31.79%	<.0001	0.1126	408	33.42%	3,801	31.79%	0.2456	0.0349	31	33.33%	737	32.11%	0.805	0.0261	48	35.82%	737	32.11%	0.3724	0.0793
High tercile	476	34.92%	3,940	32.95%	0.1429	0.0419	442	**36.20%**	3,940	32.95%	0.0217	0.069	38	40.86%	757	32.98%	0.1141	0.1671	48	35.82%	757	32.98%	0.4978	0.0602
**Comorbidity Index Scores**	**Mean**	**SD**	**Mean**	**SD**			**Mean**	**SD**	**Mean**	**SD**			**Mean**	**SD**	**Mean**	**SD**			**Mean**	**SD**	**Mean**	**SD**		
Charlson Comorbidity Index score	0.35	0.79	**0.56**	1.03	<.0001	0.207	0.3	0.69	**0.56**	1.03	<.0001	0.2526	0.39	0.77	0.63	**1.11**	0.0037	0.2239	0.37	0.93	**0.63**	1.11	0.0023	0.2361
Elixhauser Index score	1.84	1.9	**2.39**	2.32	<.0001	0.2405	1.72	1.69	**2.39**	2.32	<.0001	0.2937	1.69	1.53	2.57	**2.44**	<.0001	0.3643	2	2.09	**2.57**	2.44	0.003	0.2339
Chronic Disease Score	4.06	3.2	**4.26**	3.46	0.0243	0.0605	4.27	3.06	4.26	3.46	0.9417	0.002	4.18	3.45	4.09	3.5	0.8031	0.0264	4.36	3.28	4.09	3.5	0.3876	0.0768
**Mental Health Comorbidities**	**N**	**%**	**N**	**%**			**N**	**%**	**N**	**%**			**N**	**%**	**N**	**%**			**N**	**%**	**N**	**%**		
Depression	270	19.81%	2,602	21.76%	0.0968	0.0475	239	19.57%	2,602	21.76%	0.0767	0.0532	13	13.98%	551	**24.01%**	0.0256	0.2363	23	17.16%	551	24.01%	0.0698	0.1612
Generalized anxiety disorder	549	**40.28%**	4,165	34.83%	<.0001	0.1139	484	**39.64%**	4,165	34.83%	0.0008	0.1006	48	**51.61%**	935	40.74%	0.0367	0.221	61	45.52%	935	40.74%	0.274	0.0972
Obsessive-compulsive disorder	57	4.18%	453	3.79%	0.4733	0.0205	49	4.01%	453	3.79%	0.6962	0.0117	3	3.23%	92	4.01%	0.7049	0.04	8	5.97%	92	4.01%	0.2666	0.0987
Panic disorder	112	8.22%	922	7.71%	0.5082	0.0189	94	7.70%	922	7.71%	0.9877	0.0005	8	8.60%	206	8.98%	0.9015	0.0131	10	7.46%	206	8.98%	0.5497	0.0531
Substance use disorders	329	24.14%	3,994	**33.40%**	<.0001	0.1982	270	22.11%	3,994	**33.40%**	<.0001	0.2419	18	19.35%	668	**29.11%**	0.0416	0.2156	25	18.66%	668	**29.11%**	0.0092	0.2316
Post-traumatic stress disorder	288	21.13%	2,433	20.35%	0.4975	0.0194	211	17.28%	2,433	**20.35%**	0.0108	0.0766	20	21.51%	446	19.43%	0.6211	0.0523	22	16.42%	446	19.43%	0.3896	0.0764
**Systemic Health**	**N**	**%**	**N**	**%**			**N**	**%**	**N**	**%**			**N**	**%**	**N**	**%**			**N**	**%**	**N**	**%**		

	Patients with BPD																							
	Branded AAPs												Branded ADs											
	Copay Accumulators vs Standard Copay Plans						Copay Maximizers vs Standard Copay Plans						Copay Accumulators vs Standard Copay Plans						Copay Maximizers vs Standard Copay Plans					
	Copay Accumulators (N = 1,363)		Standard Copay Plans (N = 11,957)		P-value	Std. diff.	Copay Maximizers (N = 1,221)		Standard Copay Plans (N = 11,957)		P-value	Std. diff.	Copay Accumulators (N = 93)		Standard Copay Plans (N = 2,295)		P-value	Std. diff.	Copay Maximizers (N = 134)		Standard Copay Plans (N = 2,295)		P-value	Std. diff.
Comorbidities																								
Obesity	239	17.53%	2,488	**20.81%**	0.0045	0.0811	179	14.66%	2,488	**20.81%**	<.0001	0.1532	15	16.13%	527	22.96%	0.123	0.1632	20	14.93%	527	**22.96%**	0.0304	0.1925
Diabetes	50	3.67%	905	**7.57%**	<.0001	0.1513	24	1.97%	905	**7.57%**	<.0001	0.2193	3	3.23%	136	5.93%	0.2756	0.1153	2	1.49%	136	**5.93%**	0.0312	0.1916
Viral hepatitis	3	0.22%	180	**1.51%**	0.0001	0.1105	1	0.08%	180	**1.51%**	<.0001	0.1224	2	2.15%	24	1.05%	0.3142	0.1064	0	0.00%	24	1.05%	0.2342	0.1057
Constipation	73	5.36%	810	**6.77%**	0.0461	0.057	66	5.41%	810	6.77%	0.0674	0.055	5	5.38%	181	7.89%	0.3759	0.0936	7	5.22%	181	7.89%	0.2622	0.0996
Parkinson's disease	0	0.00%	48	**0.40%**	0.0191	0.067	3	0.25%	48	0.40%	0.4038	0.0251	0	0.00%	21	0.92%	0.3542	0.098	0	0.00%	21	0.92%	0.2661	0.0988
Chronic obstructive pulmonary disease	25	1.83%	701	**5.86%**	<.0001	0.1777	21	1.72%	701	**5.86%**	<.0001	0.1823	2	2.15%	138	6.01%	0.1201	0.1644	3	2.24%	138	6.01%	0.0693	0.1615
Chronic pain	73	5.36%	1,162	**9.72%**	<.0001	0.1506	56	4.59%	1,162	**9.72%**	<.0001	0.1774	5	5.38%	254	11.07%	0.0836	0.1831	5	3.73%	254	**11.07%**	0.0075	0.2379
Cancer	0	0.00%	5	0.04%	0.4502	0.0216	0	0.00%	5	0.04%	0.4748	0.0215	0	0.00%	1	0.04%	0.8404	0.0213	0	0.00%	1	0.04%	0.809	0.0215
Atrial fibrillation	5	0.37%	151	**1.26%**	0.0036	0.0833	5	0.41%	151	**1.26%**	0.0086	0.0789	0	0.00%	35	1.53%	0.2302	0.1269	2	1.49%	35	1.53%	0.9762	0.0027
Cardiovascular disease	12	0.88%	258	**2.16%**	0.0015	0.0907	10	0.82%	258	**2.16%**	0.0016	0.0949	2	2.15%	84	3.66%	0.4437	0.081	2	1.49%	84	3.66%	0.1869	0.1173
Peripheral vascular disease	6	0.44%	116	0.97%	0.0517	0.0556	7	0.57%	116	0.97%	0.1696	0.0413	1	1.08%	32	1.39%	0.7961	0.0273	1	0.75%	32	1.39%	0.5288	0.056

Abbreviations: AAP, atypical antipsychotics; AD, antidepressant; BPD, bipolar disease; SD, standard deviation; Std. diff., standardized difference.
